# Time series dataset of fish assemblages near thermal discharges at nuclear power plants in northern Taiwan

**DOI:** 10.1038/sdata.2018.85

**Published:** 2018-05-08

**Authors:** Hungyen Chen, Ching-Yi Chen, Kwang-Tsao Shao

**Affiliations:** 1 Department of Agronomy, National Taiwan University, Taipei 10617, Taiwan; 2 National Museum of Marine Science and Technology, Keelung 20248, Taiwan; 3 Institute of Marine Biology, National Taiwan Ocean University, Keelung 20224, Taiwan

**Keywords:** Biodiversity, Fisheries

## Abstract

Long-term time series datasets with consistent sampling methods are rather rare, especially the ones of non-target coastal fishes. Here we described a long-term time series dataset of fish collected by trammel net fish sampling and observed by an underwater diving visual census near the thermal discharges at two nuclear power plants on the northern coast of Taiwan. Both experimental and control stations of these two investigations were monitored four times per year in the surrounding seas at both plants from 2000 to 2017. The underwater visual census mainly monitored reef fish assemblages and trammel net samples monitored pelagic or demersal fishes above the muddy/sandy bottom. In total, 508 samples containing 203,863 individuals from 347 taxa were recorded in both investigations at both plants. These data can be used by ecologists and fishery biologists interested in the elucidation of the temporal patterns of species abundance and composition.

## Background & Summary

Although there are many databases for studying sustainable fishery management and marine biodiversity conservation, long-term datasets with consistent sampling methods are rather rare. To explore the possible underlying mechanism of community changes and evaluate the effectiveness of biodiversity conservation measures, databases on quantitative abundance information of consistent long-term time series are particularly important.

Two nuclear power plants (the 1st and 2nd plants) have been operating since 1970s in northern Taiwan. The construction of the 1st plant at Shihmen began in 1971 and its two generators started to operate in 1978 and 1979, respectively. The construction of the 2nd plant at Yehliu began in 1974 and its two generators started to operate in 1981 and 1983, respectively. In 2015, Chen *et al.*^1^ described a monthly impinged fish assemblage dataset containing 439 taxa over 19 years (1987–1990 and 2000–2014). They systematically collected the fish killed by impingement upon cooling water intake screens at the two nuclear power plants on the northern coast of Taiwan. In addition to the impingement investigation, which estimated fish loss because of cooling water intakes, two other investigations (underwater visual census and trammel net sampling) have been carried out to determine whether the thermal discharge have affected the local fish assemblages in the waters around the outlet area for both plants since 2000. These three investigations were conducted by the same research team and were supported by Taiwan Power Company as a long-term monitoring project to monitor fishery economics. Distinct from the monthly impinged fish assemblage dataset, underwater visual censuses and trammel net sampling were carried out four times per year because of the sea conditions, weather, and sampling cost.

In addition to the original purpose of the investigation, these experiments are also an efficient and economical way to study local fish community structure^2,3^. Where the impingement investigation provided mainly information on pelagic or non-reef associated species, underwater visual censuses primarily monitor reef fish assemblages and trammel net fishing samples monitor pelagic or demersal fishes above muddy/sandy bottoms and target species in fishery economics. It is also possible to compare the fish community structures between inlet and outlet areas of the nuclear power plants by analyzing these three datasets together.

The seas surrounding Taiwan provide a highly diverse aquatic wildlife because of complicated seafloor topography, geology, and substratum type in the surrounding offshore areas. The warm Kuroshio Current, flowing along the east coast and Asian continental shelf, is an important source for fisheries. The east coast of Taiwan lies on the deep drop-off which leads to the Philippine Sea, but the west coast is separated from China by the relatively shallow water of the Taiwan Strait. The East China Sea and cold ocean water currents flowing from the southeast coast of China in winter bring in a lot of plankton that are crucial to the continued survival of several sea organisms on the northern coast of Taiwan. As a result, studies of fish communities in the seas around Taiwan can contribute to regional fish diversity indices.

The long-term series data of fish at these two nuclear power plants in northern Taiwan have been used to study the temporal and spatial variation of fish community structure^4,5^. Chen *et al.*^1^ showed a long-term declining trend in the number of species of impinged fishes. Liao *et al.*^6^ reported that the most dominant fish at the 2nd plant changed from the high-value fish species to low-value fish species, reflecting the decline in fishery resources because of overfishing. Chen *et al.*^2^ used the impinged data to describe the altered phylogenetic species composition and proposed a phylogenetic skew index, an index of phylogenetic community diversity. Chen *et al.*^7^ showed that no significant differences were found between the fish assemblages of the thermal waters and normal ambient waters for both coral reef fishes and pelagic or demersal fishes in the surrounding seas at the 2nd plant. Chen *et al.*^3^ proposed a Bayesian hierarchical ANOVA model of stochastic seasonality to detect the annual fluctuation because of seasonality in the most dominant species, *Diodon holocanthus*, at both plants using the impinged data.

In this paper, we describe the long-term time series dataset of fish collected by trammel net fishing sampling and underwater diving visual censuses near the thermal discharges at two nuclear power plants on the northern coast of Taiwan. The time series of fish data provide a valuable resource for elucidating long-term trends in fish community ecology in this area. Additionally, these data can also be used by ecologists and fishery biologists interested in understanding the temporal pattern of species abundance and composition in relation to environmental factors, climate change, trophic interactions, and anthropogenic pressures.

## Methods

**Figure 1:**
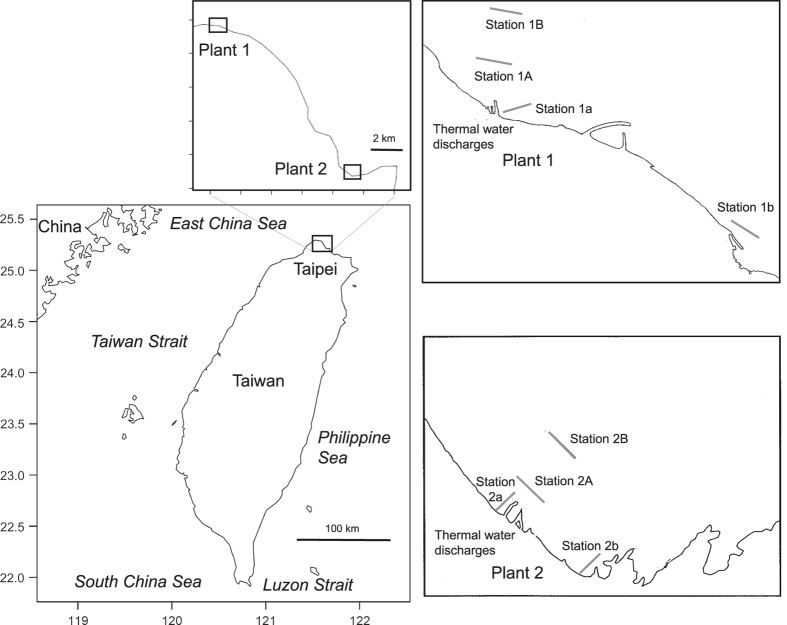
Map of sampling stations. Trammel net sampling stations (Stations 1A and 1B at Plant 1 and Stations 2A and 2B at Plant 2) and underwater visual census stations (Stations 1a and 1b at Plant 1 and Stations 2a and 2b at Plant 2) at the 1st and 2nd Nuclear Power Plants (Plants 1 and 2) in Taiwan.

The data for fish communities were collected from the sea near the thermal charges of the 1st Nuclear Power Plant at Shihmen (WGS84 N25° 17.11′, E121° 35.5′) and the 2nd Nuclear Power Plant at Yehliu (N25° 12.10′, E121° 39.46′), which is approximately 15 km east of the 1st. Both plants are situated on the northern coast of Taiwan (Fig. 1). The coastal and underwater topographies and substratum at both plants were very similar, and as such the samples collected from both plants are suggested to be comparable^1,5^. The sea floors around both plants are a mixture of coral reefs, rock (diameter <1 m), cobble, gravel, muddy/sandy patches, and large boulders. The percentage of the coverage of coral reefs, rock, and cobble is slightly higher at the area around the 1st plant than the 2nd. The sampling days were chosen by referring to meteorological conditions and avoiding sampling during the period of plant maintenance.

To monitor pelagic or demersal fishes above the muddy/sandy bottom, three-layer bottom-set trammel nets (length×height: 500 m×3 m for each layer; mesh size: 15 cm×15 cm for outer layer and 5 cm×5 cm for inner layer; material: nylon) operated by fishermen were deployed parallel at both 300 m and 800 m away from the outlet bay as the experimental (Station 1 A coordinates: N25° 17.75′, E121° 35.19′ and station 2 A coordinates: N25° 12.67′, E121° 39.96′) and control (Station 1B coordinates: N25° 18.03′, E121° 35.13′ and station 2B coordinates: N25° 12.93′, E121° 40.13′) stations, respectively, at both plants (Fig. 1). The depth of the water at the sampling stations was 18–30 m at the 1st plant and 16–25 m at the 2nd. Fish samples were collected seasonally for approximately 12 hours (from ~5 PM to ~5 AM) at the 1st plant from April 2001 (from January 2001 at Station 1A) to June 2017 and at the 2nd plant from April 2001 to June 2017.

All fish collected were brought back to the laboratory for sorting, identification, and counting. The fishes were identified personally by the fish taxonomist, Doctor Kwang-Tsao Shao (40 years of experience), and the senior laboratory member, Miss Ching-Yi Chen (20 years of experience), using numerous field guides and identification keys^8,9^. Sampling method and species identification processes were constant over the years. The identification was reliable because the number of recorded coastal species were not numerous and most of them were common species. For the rare species, specimens and DNA samples were reserved for collection and loan services.

To monitor coral reef fishes, species and abundance data were recorded by two divers (senior laboratory members having 20 years of experience, Miss Ching-Yi Chen and Mister Jeng-I Tsai) using a transect line (length: 120 m for stations 1a and 2a and 110 m for stations 1b and 2b) along the jetty of the outlet bay as the experimental station (Station 1a coordinates: N25° 17.57′, E121° 35.27′ and station 2a coordinates: N25° 12.56′, E121° 39.77′), and another jetty away from the outlet bay as the control station (Station 1b coordinates: N25° 16.99′, E121° 36.43′ and station 2b coordinates: N25° 12.18′, E121° 40.39′) at both plants (Fig. 1). The survey was carried out four times per year and continued for 40–50 min per time in the waters between 4 m and 8 m below the sea surface at the 1st plant from January 2000 (from April 2007 at Station 1b) to June 2017 and in the waters between 3 m and 6 m below the sea surface at the 2nd plant from January 2000 (from September 2000 at Station 2b) to June 2017.

Because of safety and visibility underwater, it was very difficult to measure the exact number of individuals for the schools of swimming fishes. For the schools including more than approximately 50 individuals, the number of individuals was measured by using the unit of 15/25 individuals. To verify this measuring method, underwater video was filmed to count the exact number of observed fishes later by watching the video at the laboratory. The results of counting by watching the video were very consistent with the results of the visual censuses.

The limitation of the data might be a lack of replicates at each sampling station and it was difficult to compare the seasonal variation among years^7^ (sampling months varied among years because of meteorological conditions and the periods of plant maintenance).

## Data Records

The data were represented as a list of species names and their abundance determined at both stations for each plant and each sampling method. To discourage any bad use of the data, we made four distinguished datasets including the abundance data for the two plants and the two sampling methods separately (Data Citation 1). The data collected by trammel net sampling at the 1st plant contained 124 rows (species) and 131 columns (plant, station, month, and year). The data collected by trammel net sampling at the 2nd plant contained 140 rows and 130 columns. The data collected by underwater visual censuses at the 1st plant contained 159 rows (species) and 112 columns (plant, station, month, and year). The data collected by underwater visual census at the 2nd plant contained 180 rows and 135 columns. The fish assemblage data were provided in CSV files.

## Technical Validation

**Figure 2:**
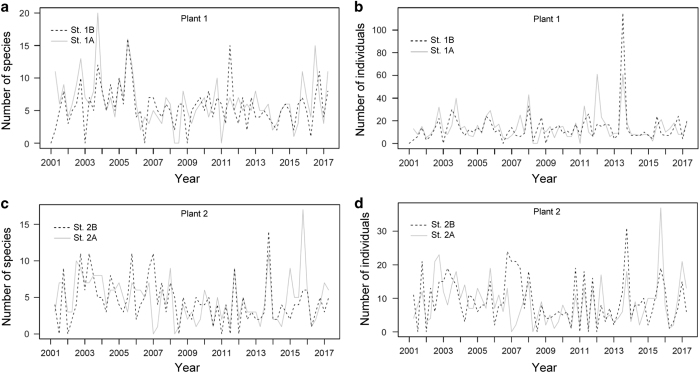
Temporal variation in the number of species and individuals collected by trammel net sampling at the 1st and 2nd Nuclear Power Plants (Plants 1 and 2) in Taiwan during the period 2001–2017. (**a**) Number of species at Plant 1. (**b**) Number of individuals at Plant 1. (**c**) Number of species at Plant 2. (**d**) Number of individuals at Plant 2.

For trammel net sampling, 1,816 individuals were collected at the 1st plant and 1,173 individuals were collected at the 2nd plant. The number of species collected per sample ranged between 0 and 20 and averaged 5.95±3.43 (s.d.) at the 1st plant and ranges between 0 and 17 and averaged 4.61±3.06 at the 2nd plant. The seasonal fluctuations in the number of species are shown in Fig. 2a and c. The number of individuals collected per sample ranged between 0 and 115 and averaged 13.86±13.41 at the 1st plant and ranged between 0 and 37 and averaged 9.02±6.80 at the 2nd plant. The seasonal fluctuations in the number of individuals are shown in Fig. 2b and d.

**Figure 3:**
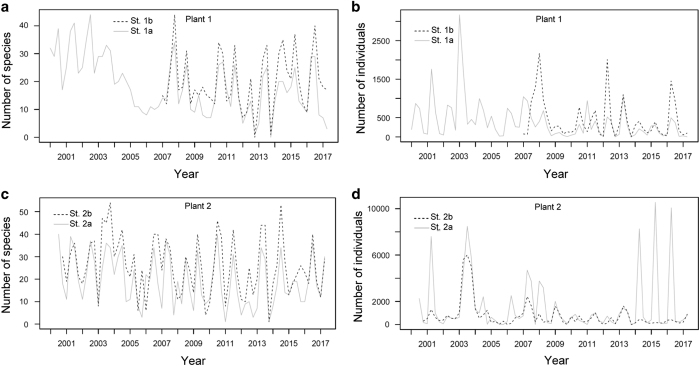
Temporal variation in the number of species and individuals observed by underwater visual censuses at the 1st and 2nd Nuclear Power Plants (Plants 1 and 2) in Taiwan during the period 2001–2017. (**a**) Number of species at Plant 1. (**b**) Number of individuals at Plant 1. (**c**) Number of species at Plant 2. (**d**) Number of individuals at Plant 2.

For underwater visual censuses, 46,219 individuals were recorded at the 1st plant and 154,655 individuals were recorded at the 2nd plant. The number of species observed per sample ranges between 0 and 44 and averaged 19.11±10.18 at the 1st plant and ranges between 1 and 54 and averaged 22.60±12.37 at the 2nd plant. The seasonal fluctuations in the number of species are shown in Fig. 3a and c. The number of individuals observed per sample ranged between 0 and 3,170 and averaged 412.67±510.61 at the 1st plant and ranged between 1 and 10,550 and averaged 1145.59±1957.84 at the 2nd plant. The seasonal fluctuations in the number of individuals are shown in Fig. 3b and d.

Figures 2 and 3 show a slightly decreasing trend in the number of species during the period from 2001 to 2014 at both plants, especially in the trammel net samples. For the trammel net samples, their fluctuations in the number of species became more moderate after 2007 at both plants (Fig. 2a and c), which was caused by the disappearance of the seasonal species. The resident fish appeared steadily, but the number of migrant fish declined. Because the sampling method and species identification process were constant over years, artificial factors should be excluded from the causes of the changed pattern.

**Figure 4:**
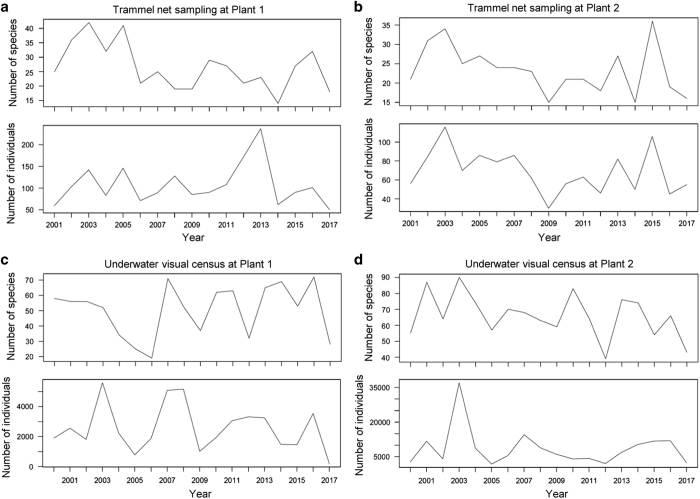
Yearly variations in the numbers of species and individuals recorded by trammel net sampling and underwater visual censuses at the 1st and 2nd Nuclear Power Plants (Plants 1 and 2) in Taiwan during the period 2000–2017. (**a**) Trammel net sampling at Plant 1. (**b**) Trammel net sampling at Plant 2. (**c**) Underwater visual censuses at Plant 1. (**d**) Underwater visual censuses at Plant 2.

To see the yearly variations, we merged the samples in each year and calculated yearly numbers of species and individuals from both sampling methods at both plants (Fig. 4). In the trammel net samples, a slightly decreasing yearly trend in the number of species was found during the period from 2001 to 2014 at both plants.

## Additional information

**How to cite this article:** Chen H. *et al.* Time series dataset of fish assemblages near thermal discharges at nuclear power plants in northern Taiwan. *Sci. Data* 5:180085 doi: 10.1038/sdata.2018.85 (2018).

**Publisher’s note:** Springer Nature remains neutral with regard to jurisdictional claims in published maps and institutional affiliations.

## Supplementary Material
